# In Patients With Severe Alcoholic Hepatitis, Prednisolone Increases Susceptibility to Infection and Infection-Related Mortality, and Is Associated With High Circulating Levels of Bacterial DNA

**DOI:** 10.1053/j.gastro.2016.12.019

**Published:** 2017-04

**Authors:** Nikhil Vergis, Stephen R. Atkinson, Suzanne Knapp, James Maurice, Michael Allison, Andrew Austin, Ewan H. Forrest, Steven Masson, Anne McCune, David Patch, Paul Richardson, Dermot Gleeson, Stephen D. Ryder, Mark Wright, Mark R. Thursz

**Affiliations:** 1 Imperial College, London, United Kingdom; 2 Addenbrookes Hospital, Cambridge, United Kingdom; 3 Derby Hospital, Derby, United Kingdom; 4 Glasgow Royal Infirmary, Glasgow, United Kingdom; 5 Freeman Hospital, The Newcastle Upon Tyne Hospitals, National Health Service Foundation Trust, Newcastle Upon Tyne, United Kingdom; 6 Bristol Royal Infirmary, Bristol, United Kingdom; 7 Royal Free Hospital, London, United Kingdom; 8 Royal Liverpool University National Health Service Trust, Liverpool, United Kingdom; 9 Sheffield Teaching Hospitals Foundation Trust, Sheffield, United Kingdom; 10 NIHR Biomedical Research Unit in Gastrointestinal and Liver Diseases at Nottingham University Hospitals National Health Service Trust and The University of Nottingham, Nottingham, United Kingdom; 11 Southampton University Hospital, Southampton, United Kingdom

**Keywords:** STOPAH Trial, MELD, *E coli*, Steroid, bDNA, bacterial DNA, CI, confidence interval, ^hi^bDNA, bacterial DNA >18 pg/mL, DF, discriminant function, MELD, Model for End-Stage Liver Disease, OR, odds ratio, PCR, polymerase chain reaction, SAE, serious adverse event, SAH, severe alcoholic hepatitis, STOPAH, Steroids or Pentoxifylline for Alcoholic Hepatitis

## Abstract

**Background & Aims:**

Infections are common in patients with severe alcoholic hepatitis (SAH), but little information is available on how to predict their development or their effects on patients. Prednisolone is advocated for treatment of SAH, but can increase susceptibility to infection. We compared the effects of infection on clinical outcomes of patients treated with and without prednisolone, and identified risk factors for development of infection in SAH.

**Methods:**

We analyzed data from 1092 patients enrolled in a double-blind placebo-controlled trial to evaluate the efficacy of treatment with prednisolone (40 mg daily) or pentoxifylline (400 mg 3 times each day) in patients with SAH. The 2 × 2 factorial design led to 547 patients receiving prednisolone; 546 were treated with pentoxifylline. The trial was conducted in the United Kingdom from January 2011 through February 2014. Data on development of infection were collected at evaluations performed at screening, baseline, weekly during admission, on discharge, and after 90 days. Patients were diagnosed with infection based on published clinical and microbiologic criteria. Risk factors for development of infection and effects on 90-day mortality were evaluated separately in patients treated with prednisolone (n = 547) and patients not treated with prednisolone (n = 545) using logistic regression. Pretreatment blood levels of bacterial DNA (bDNA) were measured in 731 patients.

**Results:**

Of the 1092 patients in the study, 135 had an infection at baseline, 251 developed infections during treatment, and 89 patients developed an infection after treatment. There was no association between pentoxifylline therapy and the risk of serious infection (*P* = .084), infection during treatment (*P* = .20), or infection after treatment (*P* = .27). Infections classified as serious were more frequent in patients treated with prednisolone (odds ratio [OR], 1.27; 95% confidence interval [CI], 1.27−2.92; *P* = .002). There was no association between prednisolone therapy and infection during treatment (OR, 1.04; 95% CI, 0.78−1.37; *P* = .80). However, a higher proportion (10%) of patients receiving prednisolone developed an infection after treatment than of patients not given prednisolone (6%) (OR, 1.70; 95% CI, 1.07−2.69; *P* = .024). Development of infection was associated with increased 90-day mortality in patients with SAH treated with prednisolone, independent of model for end-stage liver disease or Lille score (OR, 2.46; 95% CI, 1.41−4.30; *P* = .002). High circulating bDNA predicted infection that developed within 7 days of prednisolone therapy, independent of Model for End-Stage Liver Disease and white blood cell count (OR, 4.68; 95% CI, 1.80−12.17; *P* = .001). In patients who did not receive prednisolone, infection was not independently associated with 90-day mortality (OR, 0.94; 95% CI, 0.54−1.62; *P* = .82) or levels of bDNA (OR, 0.83; 95% CI, 0.39−1.75; *P* = .62).

**Conclusions:**

Patients with SAH given prednisolone are at greater risk for developing serious infections and infections after treatment than patients not given prednisolone, which may offset its therapeutic benefit. Level of circulating bDNA before treatment could identify patients at high risk of infection if given prednisolone; these data could be used to select therapies for patients with SAH. EudraCT no: 2009-013897-42; Current Controlled Trials no: ISRCTN88782125.

See editorial on page 938.

Severe alcoholic hepatitis (SAH) is a clinical syndrome characterized by the recent onset of jaundice and liver failure after prolonged, heavy alcohol misuse. Severe cases are defined by the Maddrey’s discriminant function (DF), a calculation utilizing the serum bilirubin and prothrombin time. Where DF is ≥32, ninety-day mortality is 30%−40%; below this threshold spontaneous survival is >95%.^1^, ^2^, ^3^ In common with other forms of liver failure, SAH is associated with increased susceptibility to infection. In the context of SAH, it has been reported that 13%–25% of patients have an infection at presentation and a similar proportion develop an infection during treatment.^3^, ^4^

Current guidelines recommend the use of prednisolone, a corticosteroid with broad anti-inflammatory and immunosuppressive actions for the management of SAH, although few studies have shown benefit beyond 28 days.^5^, ^6^, ^7^ In the Steroids or Pentoxyfilline for Alcoholic Hepatitis (STOPAH) trial, prednisolone almost doubled the risk of infections reported as serious adverse events (13% vs 7%, which was significant at the *P* = .002 level). However, the relationships between prednisolone and liver function, infection, and mortality remain contentious.^4^, ^8^

The aim of this study was to characterize the incidence and impact of infection in SAH using the data from the large cohort of patients recruited to the multicenter STOPAH trial. In addition, this study evaluates pretreatment circulating levels of 16S ribosomal bacterial DNA (bDNA) as a predictor of the subsequent development of infection in patients treated with and without prednisolone by random double-blind allocation.

## Materials and Methods

### Study Population

Patients were recruited in accordance with the STOPAH trial protocol.^9^ All had a history of alcohol misuse; compatible clinical, laboratory, and/or liver biopsy features of alcoholic hepatitis; no other identified causes of liver disease; and DF ≥32. Infections, if present, were treated and controlled with antibiotics before enrolment. All participants, or their legally appointed representative, provided written informed consent.

The trial was approved by the Multicenter Research Ethics Committee (reference 09/MRE09/59) and conducted in accordance with the Medicines for Human Use (Clinical Trials) Regulations 2004 (2006 amendment); the European Union Clinical Trials Directive (Directive 2001/20/EC) guidelines; the principles of the International Conference on Harmonization Good Clinical Practice and under the oversight of University of Southampton Clinical Trials Unit. All participants, or their legally appointed representative, provided written informed consent. All authors had access to the study data and have reviewed and approved the final manuscript.

### Group Allocation

STOPAH utilized a double-blind, double-dummy, 2 × 2 factorial design.^9^ Patients were randomized to treatment with 40 mg prednisolone once a day or 400 mg pentoxyfilline 3 times a day, neither, or both. There was no mortality benefit from pentoxyfilline, but a possible 28-day mortality benefit from prednisolone.^10^ The effect of prednisolone on infection was examined by comparing 2 groups: prednisolone (n = 547) and no-prednisolone−treated patients (n = 545).

### Mortality Data

Data regarding date and cause of death were collected during the follow-up period. Patients were also consented for follow-up via the National Health Service Information Centre Data Linkage service, ensuring that if they were lost to follow-up and died, this information could be captured. Mortality at 90 days was analyzed in order to capture the occurrence and impact of all infections occurring during or after the treatment period.

### Periods of Infection and Antibiotic Treatment

Clinical data regarding the development of infection were collected at trial visits that occurred at screening, baseline, weekly during admission, on discharge and at 90 days. Data regarding the development of infection submitted in reports of serious adverse events (SAEs) were also incorporated. The diagnosis of infection was made prospectively by treating physicians who were blind to treatment allocation with or without prednisolone. Diagnosis was guided by criteria for infection in the setting of liver disease outlined by Bajaj et al.^11^

Baseline infections were defined as those that occurred between admission and the start of therapy. Active antibiotic treatment at the start of trial therapy was defined as intravenous antibiotics commenced and continued within 5 days prior to treatment start date. Incident infections were defined as those that occurred after the start of treatment—these were further broken down into 3 categories relevant to the clinical management of these patients:

1. Day 7 infections occurred within the first 7 days of therapy (aligned with liver function data available at 7 days from which Lille score was calculated);
2. On-treatment infections within the study treatment period (28 days);
3. Post-treatment infections occurring in the day 28 to day 90 follow-up period;

### Bacterial DNA Measurement

An EDTA blood sample was taken from patients at enrolment. DNA extraction was performed on 400 μL blood using Qiagen (Hilden, Germany) QIAamp DNA Mini kits under aseptic conditions. The quantity of 16S ribosomal bDNA was determined and measured by real-time polymerase chain reaction (PCR). There are no established cut-off values that define positive from negative bDNA values. In this study, bDNA level that had 80% specificity for predicting the subsequent development of infection in prednisolone-treated patients within 7 days (18.5 pg/mL) was considered a high bDNA level (^hi^bDNA) for subsequent modeling analyses.

The PCR methodology was adapted from that reported previously.^12^ Briefly, primers directed against the V7−V9 variable region of the 16S gene (forward: RW01; 5′->3′ sequence AACTGGAGGAAGGTGGGGAT, reverse: DG74.R; 5′->3′ sequence AGGAGGTGATCCAACCGCA) were combined with a custom fluorescent probe (6-FAM- TACAAGGCCCGGGAACGTATTCACCG-TAMRA; Life Technologies, Carlsbad, CA) at final concentrations of 0.5 μM and 0.25 μM, respectively. This was combined with 10 μL Taqman Gene Expression mix (Applied Biosciences, Foster City, CA), 4 μL extracted DNA and PCR-grade water, to give a final reaction volume of 20 μL. PCR was performed on a StepOne Plus PCR machine (Applied Biosciences) with hot-start activation (2 minutes at 50°C, 10 minutes at 95°C) and 40 reaction cycles (15 seconds at 95°C, 30 seconds at 60°C and 60 seconds at 72°C to collect fluorescence). Serial 10-fold dilutions of *Escherichia coli* DNA (0.08 ng/μL to 0.000008 ng/μL) and a negative control were run to generate a standard curve. Standards and samples were run in triplicate. Any sample displaying a positive signal at or below the level of the negative control was considered negative. Any triplicate group with readings >1 copy cycle apart was considered unreliable and discarded; otherwise, the mean reading was calculated. Standard curves were generated and concentrations interpolated in Prism, version 7.0 (GraphPad, La Jolla, CA). bDNA levels are given as picograms bDNA per milliliter of whole blood from which it was extracted.

### Statistical Analysis

Statistical analyses were conducted in SPSS, version 23 (IBM, Armonk, NY) and survival curves were drawn using R (Vienna, Austria). Comparisons between groups were tested using either Mann−Whitney U test for nonparametrically distributed continuous variables or χ^2^ test for proportions. Associations between explanatory variables and end points were tested using logistic regression. Early improvement in liver function was defined as Lille score <.45.^13^

In light of previously published data regarding the relationship between prednisolone and early improvement in liver function, infection, and mortality,^4^ we tested, a priori, for an interaction between these factors and the end points under consideration by logistic regression.

Previous studies have confirmed that infection and mortality, if present, are positively associated.^4^, ^8^ In view of this, and the biologic implausibility that infection could be associated with reduced mortality, a one-tailed test of association between bDNA and 90-day mortality in prednisolone-treated patients was performed. Secondary outcomes were tested post hoc and are not corrected for multiple testing because they are exploratory. For analyses that modeled the expected 90-day mortality in patients with high bDNA treated with or without prednisolone, matching was performed using the FUZZY extension within SPSS, specifying tolerance of 2 pg/mL bDNA.

## Results

### Population Characteristics

Data regarding infection were available in 1092 of 1103 (99%) of patients randomized in the STOPAH trial; baseline characteristics are presented in Table 1.

**Table 1:** Baseline Characteristics of Study Population

Variable	All patients	Baseline infection only (n = 94)	Baseline and incident infection (n = 41)	Incident infection only (n = 268)	Never infected (n = 689)
Age, *y*	48.8 (41.9−56.3)	49.5 (41.9−54.7)	47.1 (41.1−56.9)	50.3 (42.6−58.8)	48.3 (41.8−55.8)
Sex, male, n (*%*)	685 (62.7)	60 (63.8)	26 (63.4)	159 (59.3)	440 (63.9)
Alcohol consumption, *U/wk*	132 (84−210)	125 (80−197)	184 (96−249)	120 (80−199)	128 (84−210)
Prednisolone, n (*%*)	547 (50)	44 (47)	20 (49)	144 (54)	339 (49)
Systolic blood pressure, *mm Hg*	110 (102−120)	112 (105−121)	113 (100−126)	110 (100−120)	110 (102−120)
Diastolic blood pressure, *mm Hg*	90 (60−74)	69 (60−77)	66 (58−77)	65 (60−73)	68 (60−75)
Pulse, *beats/min*	90 (80−98)	82 (88−98)	95 (77−102)	91 (80−100)	89 (80−98)
Temperature, *°C*	36.8 (36.5−37.1)	36.8 (36.6−37.1)	36.8 (36.4−37.3)	36.8 (36.5−37.1)	36.8 (36.5−37.1)
Hemoglobin, *g/L*	107 (94−120)	102 (90−114)	100 (88−118)	105 (94−120)	108 (95−121)
Total white cell count, *×10*^*3*^*per mm*^*3*^	9.00 (6.23−12.6)	9.90 (6.68−14.4)	10.6 (7.05−16.1)	10.1 (7.1−13.7)	8.20 (6.00−11.9)
Neutrophils, *×10*^*3*^*per mm*^*3*^	6.2 (4.1−9.8)	7.2 (4.2−11.6)	6.9 (5.4−13.3)	7.3 (4.9−11.0)	5.7 (3.9−9.0)
International normalized ratio	1.80 (1.56−2.09)	1.91 (1.60−2.32)	1.74 (1.58−2.00)	1.82 (1.60−2.12)	1.70 (1.51−2.00)
Albumin, g*/L*	25 (21−29)	26 (22−31)	25 (18−31)	24 (20−28)	25 (21−29)
Bilirubin, *mg/dL*	16.1 (10.1−24.4)	14.7 (9.47−24.4)	18.6 (9.6−25.7)	16.7 (10.6−25.1)	15.9 (9.90−24.0)
Alanine transaminase, *IU/L*	43 (30−61)	38 (27−51)	39 (31−61)	44 (28−64_	43 (31−62)
Aspartate transaminase, *IU/L*	124 (87−169)	125 (89−148)	120 (90−164)	122 (87−178)	125 (87−171)
Sodium, *mmol/L*	134 (130−136)	134 (131−138)	134 (130−137)	133 (130−136)	134 (130−137)
Urea, *mmol/L*	3.3 (2.2−5.2)	3.5 (2.4−6.7)	4.3 (2.6−7.1)	3.6 (2.2−5.4)	3.1 (2.2−4.9)
Creatinine, *mg/dL*	0.72 (0.60−0.97)	0.72 (0.59−0.99)	0.75 (0.62−1.03)	0.76 (0.60−1.06)	0.72 (0.60−0.92)
Discriminant function^a^	55.4 (43.1−73.7)	62.1 (46.6−86.7)	56.9 (47.0−68.4)	60.6 (45.5−82.0)	53.4 (42.1−69.8)
Model for End-Stage Liver Disease^b^	23.4 (21.0−26.4)	24.4 (21.7−28.6)	24.3 (21.7−27.0)	24.2 (21.4−28.1)	22.9 (20.8−25.7)

NOTE. Groupings are based on the entire study population, with subgroups of when the infection was diagnosed relative to the start of treatment. Baseline infection was defined as those that occurred between admission and the start of therapy. Incident infections were those that occurred after initiation of therapy. Data are presented median (interquartile range) unless otherwise indicated.

a Discriminant function = 4.6 × (PT_Patient_ − PT_Control_ [seconds]) + bilirubin [mg/dL].

b Model for End-Stage Liver Disease = 3.78 × ln[serum bilirubin (mg/dL)] + 11.2 × ln[INR] + 9.57 × ln[serum creatinine (mg/dL)] + 6.43.

### Baseline Infection

Infection at baseline occurred in 12% (135 of 1092) of patients (Supplementary Table 1). Chest infections were the single largest category, accounting for 34% (42 of 125) of infections that specified a site of origin (Supplementary Table 2). Positive microbiological cultures were reported in 56 of 135 (41%) patients. *E coli* was the most commonly isolated organism (12 of 40 [30%]; Supplementary Table 3).

Between admission and initiation of trial therapy, 492 of 1092 (45%) patients were prescribed an antibiotic. Of those patients, 293 (60%) continued to receive antibiotic therapy into the treatment period.

**Figure 1:**
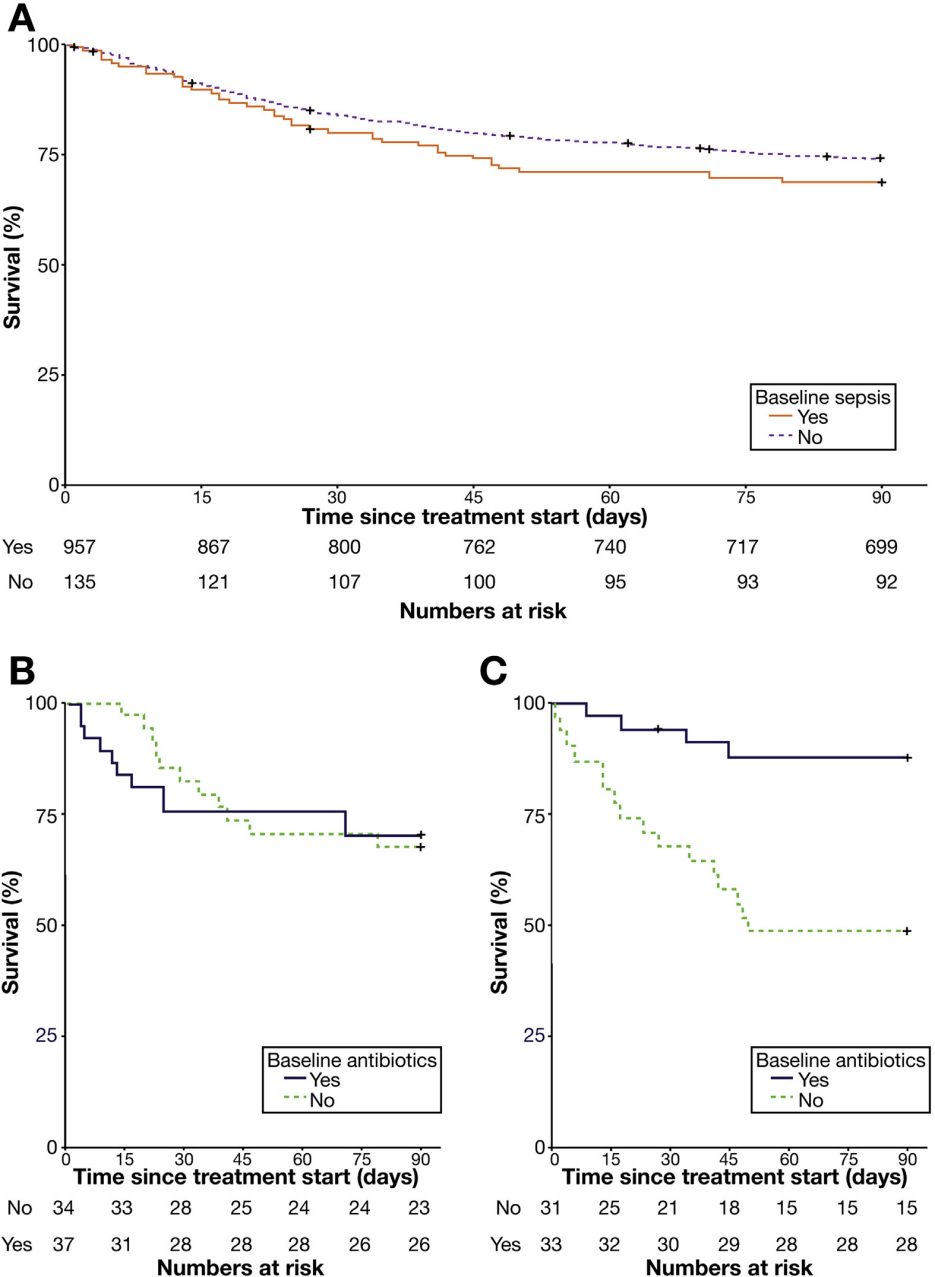
Prescription of antibiotics significantly modulates the impact of baseline infection on 90-day mortality in prednisolone-treated patients. In all patients, no statistically significant impact of baseline sepsis on mortality is seen (*A*). In patients who present with infection and do not receive prednisolone, continuation of antibiotics alongside treatment for AH does not impact upon mortality (*B*), however, in patients who receive prednisolone concurrent antibiotic therapy significantly reduces mortality (*C*).

Overall, there was no statistically significant association between baseline infection and mortality at 90 days (31% vs 26%; odds ratio [OR], 1.31; 95% confidence interval [CI], 0.88−1.94; *P* = .18; Figure 1*A*). In patients with baseline infection who did not receive prednisolone, active antibiotic therapy when starting treatment had no impact on mortality (30% vs 32%; *P* = .81; Figure 1*B*). However, in those who received prednisolone, there was a significant reduction in 90-day mortality associated with continued antibiotic therapy when compared with those patients in whom antibiotic therapy was stopped before initiating prednisolone (13% vs 52%; OR, 0.13; 95% CI, 0.038−0.47; *P* = .002; Figure 1*C*).

### Incident Infection

On-treatment infections were diagnosed in 251 patients (23%) and post-treatment infections were seen in 89 patients (Supplementary Table 1). The most common site of infection in both cases was chest (37% [110 of 301] and 39% [40 of 102], respectively). On-treatment infection was significantly associated with recurrent post-treatment infection risk (OR, 1.93; 95% CI, 1.21−3.06; *P* = .005).

Taken together positive cultures were reported in 147 of 372 episodes of incident infection (40%). *E coli* was the most frequently cultured organism (33 of 133 [25%]; Supplementary Table 3). In patients developing incident infection, median time to develop the infection was 13 days after the start of treatment.

Univariable factors associated with the development of incident infection are given in Table 2. On multivariable analysis, an independent effect was demonstrated for peripheral white cell count (OR, 1.04; 95% CI, 1.02−1.07; *P* = .002) and age (OR, 1.02; 95% CI, 1.00−1.03; *P* = .01). Baseline DF and Model for End-Stage Liver Disease (MELD) scores were both strongly associated with the subsequent risk of developing an infection (*P* = .002 and *P* < .001, respectively; Table 2).

**Table 2:** Associations Between Baseline Characteristics and the Development of Incident Infection

Variable	Univariable		Multivariable	
	OR (95% CI)	*P* value	OR (95% CI)	*P* value
Demographics				
Age, *y*	1.01 (1.00−1.03)	.055	1.02 (1.00−1.03)	.013
Sex, *male*	1.18 (0.90−1.55)	.220	—	—
Alcohol consumption, *U/wk*	1.00 (0.99−1.00)	.522	—	—
Observations				
Systolic blood pressure, *mm Hg*	1.00 (0.99−1.01)	.909	—	—
Diastolic blood pressure, *mm Hg*	0.99 (0.98−1.00)	.098	0.99 (0.98−1.00)	.177
Pulse, *beats/min*	1.01 (0.99−1.02)	.077	1.01 (1.00−1.02)	.056
Temperature, *°C*	1.06 (0.82−1.39)	.656	—	—
Hematology and biochemistry				
Hemoglobin, *g/L*	0.99 (0.99−1.00)	.221	—	—
Total WBC, *×10*^*3*^*per mm*^*3*^	1.05 (1.03−1.08)	<.001	1.04 (1.02−1.07)	.002
Neutrophils, *×10*^*3*^*per mm*^*3*^	1.06 (1.03−1.08)	<.001	—	—
INR	1.45 (1.12−1.89)	.005	1.31 (0.99−1.73)	.058
Albumin, *g/L*	0.98 (0.95−0.99)	.031	0.98 (0.96−1.00)	.092
Bilirubin, *mg/dL*	1.01 (0.99−1.03)	.065	1.00 (0.99−1.02)	.648
Alanine transaminase, *IU/L*	1.00 (0.99−1.00)	.841	—	—
Aspartate transaminase, *IU/L*	0.99 (0.99−1.00)	.485	—	—
Sodium, *mmol/L*	0.98 (0.95−1.00)	.062	0.99 (0.97−1.02)	.695
Urea, *mmol/L*	1.03 (0.99−1.06)	.102	—	—
Creatinine, *mg/dL*	1.38 (1.09−1.75)	.009	1.20 (0.91−1.58)	.203
Clinical scores				
Discriminant function^a^	1.01 (1.00−1.01)	.002	—	—
MELD^b^	1.06 (1.03−1.09)	<.001	—	—

NOTE. Variables showing a trend to significance on univariable analysis (*P* < .10) were entered into multivariable analysis.

INR, international normalized ratio; MELD, Model for End-Stage Liver Disease; WBC, white blood cell count.

a Discriminant function = 4.6 × (PT_Patient_-PT_Control_ [seconds]) + bilirubin [mg/dL].

b MELD = 3.78 × ln[serum bilirubin (mg/dL)] + 11.2 × ln[INR] + 9.57 × ln[serum creatinine (mg/dL)] + 6.43

### Treatment and Infection Risk

Serious infections (SAEs), on-treatment infections, and post-treatment infections were considered separately when testing for associations with treatment, in light of published findings that prednisolone increases the risk of serious and late infections in particular.^3^, ^14^

### Pentoxyfilline

There was no association between pentoxyfilline therapy and the risk of serious (SAE), on-treatment, or post-treatment infections (OR, 0.70; 95% CI, 0.46−1.05; *P* = .084; OR, 0.83; 95% CI, 0.63−1.10; *P* = .20; and OR, 0.78; 95% CI, 0.50−1.21; *P* = .27, respectively).

### Prednisolone

Infections classified as serious (SAEs) were more frequent in patients treated with prednisolone (OR, 1.27; 95% CI, 1.27−2.92; *P* = .002).^3^ There was no association between prednisolone therapy and on-treatment infection (OR, 1.04; 95% CI, 0.78−1.37; *P* = .80). However, prednisolone was associated with an increased risk of developing post-treatment infection (56 of 547 [10%] vs 33 of 545 [6%]; OR, 1.70; 95% CI, 1.07−2.69; *P* = .024).

In addition, there were significant interactions between prednisolone and Lille response in relation to both 90-day mortality (*P* = .00017) and infection (*P* = .045). Consequently, prednisolone and no-prednisolone groups were considered separately for statistical analyses other than comparisons between treatment arms.

Development of an incident infection was significantly associated with mortality in prednisolone-treated patients (prednisolone: 39% vs 22%; OR, 2.27; 95% CI, 1.52−3.38; *P* < .0001), but was not in the patients who did not receive prednisolone (31% vs 24%; OR, 1.36; 95% CI, 0.89−2.08; *P* = .15).

Multivariable analysis incorporating terms reflecting development of infection, baseline severity of liver disease (MELD), presence of encephalopathy, and response to treatment (Lille score <0.45) was performed. In prednisolone-treated patients an independent effect of infection on 90-day mortality was seen (OR, 2.46; 95% CI, 1.41−4.30; *P* = .002) (Table 3).

**Table 3:** Multivariable Analysis Examining the Effect of Incident Infection on Mortality by Logistic Regression, After Adjusting Liver Function (Model for End-Stage Liver Disease), Encephalopathy, and Treatment Response (Lille Response)

Variable	Prednisolone		No prednisolone	
	OR (95% CI)	*P* value	OR (95% CI)	*P* value
Infection	2.46 (1.41−4.30)	.002	.94 (.54−1.62)	.82
MELD	1.08 (1.02−1.15)	.012	1.12 (1.06−1.20)	<.001
Encephalopathy	1.83 (1.02−3.28)	.042	2.19 (1.24−3.84)	.007
Lille response	.36 (.21−.64)	<.001	.29 (.16−.50)	<.001

NOTE. Results are given for both prednisolone-treated and no-prednisolone groups.

MELD, Model For End-Stage Liver Disease.

### Alcohol and Infection Risk

Recidivism after the episode of SAH was recorded at 90 days. Importantly, there was no association between prednisolone treatment and a return to alcohol drinking (*P* = .95). Further detail is provided in Supplementary Results.

### Infection and Early Improvement in Liver Function

Failure to demonstrate an early improvement in liver function (Lille score >.45) was associated with an increased risk of infection in prednisolone-treated patients (52% vs 29%; OR, 2.70; 95% CI, 1.69−4.32; *P* = .00003), but not in patients treated without prednisolone (34% vs 29%; OR, 1.28; 95% CI, 0.82−1.98; *P* = .28).

**Figure 2:**
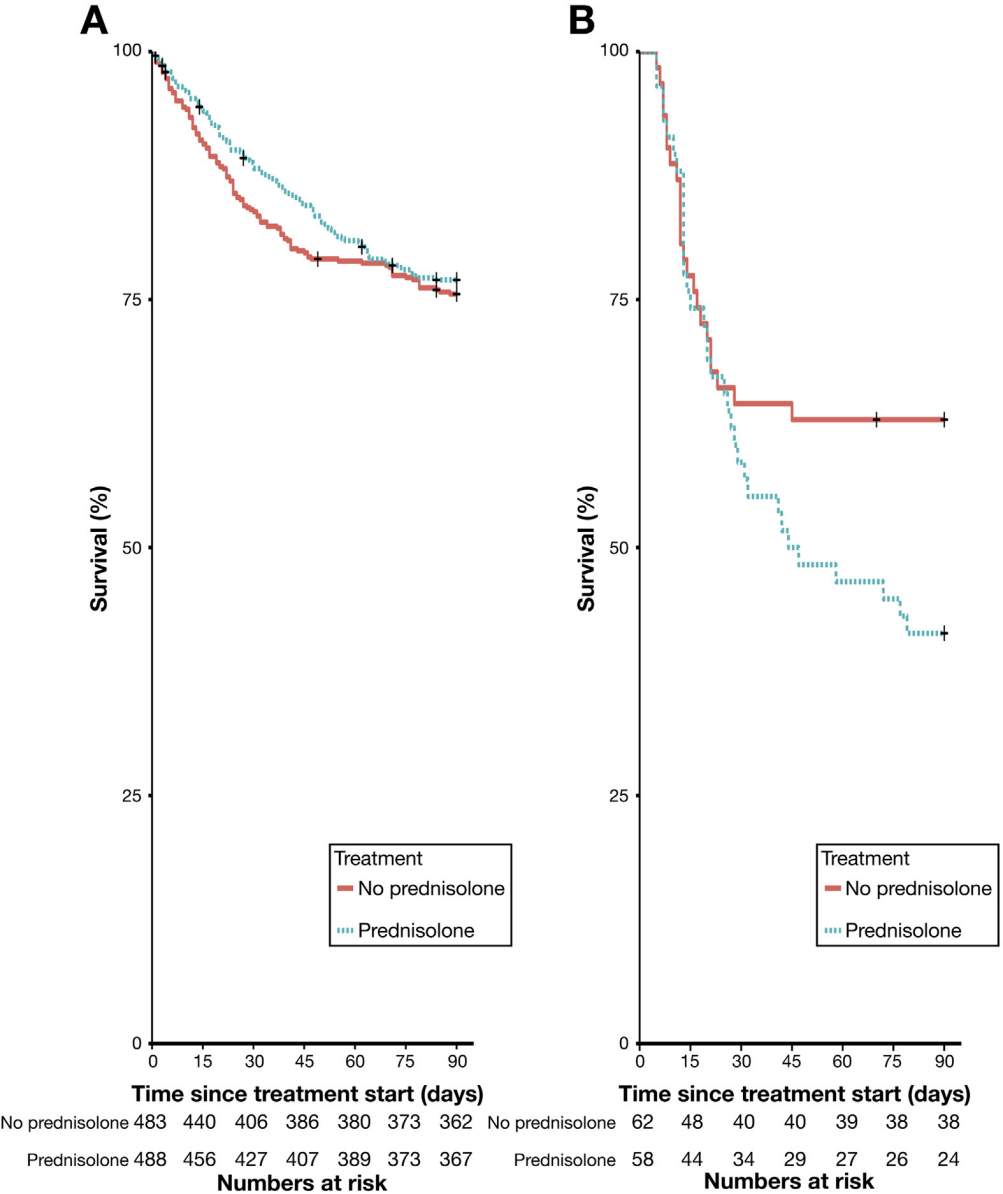
Early-onset infection leads to excess mortality in patients treated with prednisolone. In patients who do not develop infection within the first 7 days, there is a nonsustained improvement in mortality at 28 days (*A*). However, in patients who have early onset of infection, treatment with prednisolone is associated with a dramatic increase in mortality (*B*).

Day 7 infections, developing before calculation of the Lille score at day 7, were associated with a significantly increased risk of Lille nonresponse in prednisolone-treated patients (OR, 2.82; 95% CI 1.48−5.26; *P* = .002), but not in patients treated without prednisolone (OR, 1.28; 95% CI, 0.70−2.34; *P* = .43). Accordingly, prednisolone treatment was associated with a significant increase in 90-day mortality in patients who developed infection within 7 days (59% vs 38%; OR, 2.34; 95% CI, 1.12−4.88; *P* = .023) (Figure 2).

### Utility of Bacterial DNA Level to Predict Infection and Mortality

Whole blood samples were available for bDNA analysis in 68% (740 of 1092) of patients included in the clinical data analysis. Further detail regarding characteristics of patients from whom bDNA results were not available is provided in Supplementary Results.

Ninety percent of SAH patients (661 of 731) had detectable bDNA from whole blood samples. However, there was no correlation between age or alcohol consumption and bDNA (*r*_s_ < −.01, *P* = .97 and *r*_s_ = −.05, *P* = .21). There was also no correlation between baseline bDNA and baseline liver function as described by MELD, DF, or Glasgow Alcoholic Hepatitis Score (*r*_s_ = .04, *P* = .25; *r*_s_ = .04, *P* = .25; and *r*_s_ = .04, *P* = .32, respectively). Clinical characteristics of patients are presented in Supplementary Table 4 by day 7 infection status.

Because antibiotic therapy before sampling is likely to reduce bDNA levels, we sought and found an interaction between bDNA and intravenous antibiotic therapy in the prediction of day 7 infection (*P* = .02). Patients who had been treated with intravenous antibiotics within 5 days before sampling were therefore excluded (195 patients, leaving 536 patients available for further analysis). Patients were further divided by treatment with prednisolone (prednisolone, n = 265; no prednisolone n = 271) in line with previous analyses.

There was a striking association between bDNA and development of infection within 7 days in patients treated with prednisolone (developed infection vs did not develop infection: 20.9 vs 8.3 pg/mL [median values]; *P* = .004). Area under receiver operating characteristic curve for prednisolone-treated patients was .704 (95% CI .58−.83; *P* = .0032). By way of comparison, the area under the receiver operating characteristic for white blood cell count to predict infection within 7 days was .577, but this was not statistically significant (*P* = .265; Supplementary Table 5). bDNA level was not associated with day 7 infection in patients treated without prednisolone (developed infection vs did not develop infection: 12.7 vs 12.3 pg/mL; *P* = .95).

A cut-off of 18.5 pg/mL bDNA was 80% specific for prediction of infection within 7 days. This cut-off was used to define a high level of bDNA (^hi^bDNA). ^hi^bDNA was associated with increased risk of infection by day 7 in prednisolone-treated patients (OR, 4.48; 95% CI, 1.70−11.81; *P* = .002). This association remained significant after multivariable analysis that controlled for confounding factors of MELD and white blood cell count (Table 4). In contrast, ^hi^bDNA was not associated with the development of day 7 infection in either univariable or multivariable analysis for patients treated without prednisolone (Table 4).

**Table 4:** Multivariable Logistic Regression Analysis Incorporating Bacterial DNA, Model for End-Stage Liver Disease, and White Blood Cell Count for Prediction of Day 7 Infection in Patients Treated With and Without Prednisolone

Variable	Prednisolone		No prednisolone	
	OR (95% CI)	*P* value	OR (95% CI)	*P* value
^hi^bDNA	4.68 (1.80−12.17)	.001	0.83 (0.39−1.75)	.62
MELD	1.08 (0.99−1.17)	.097	1.07 (0.99−1.15)	.08
WBC	1.06 (0.97−1.16)	.187	1.07 (0.99−1.15)	.07

MELD, Model for End-Stage Liver Disease; WBC, white blood cell count.

All patients were considered for survival analyses (n = 731). bDNA level before treatment correlated with Lille score (*r*_s_ = .16; *P* = .0006), irrespective of antibiotic treatment (*r*_s_ = .27, *P* = .003 for antibiotic treated patients and *r*_s_ = .12, *P* = .02 for patients not treated with antibiotics within 5 days before sampling). In addition, bDNA level was higher for patients who died by 90 days compared with those who survived to 90 days (11.2 vs 9.3 pg/mL; *P* = .04). ^hi^bDNA was associated with 90-day mortality (OR, 1.39; 95% CI, .98−2.0; 29% vs 23% 90-day mortality in patients with ^hi^bDNA vs patients without ^hi^bDNA; *P* = .03).

**Figure 3:**
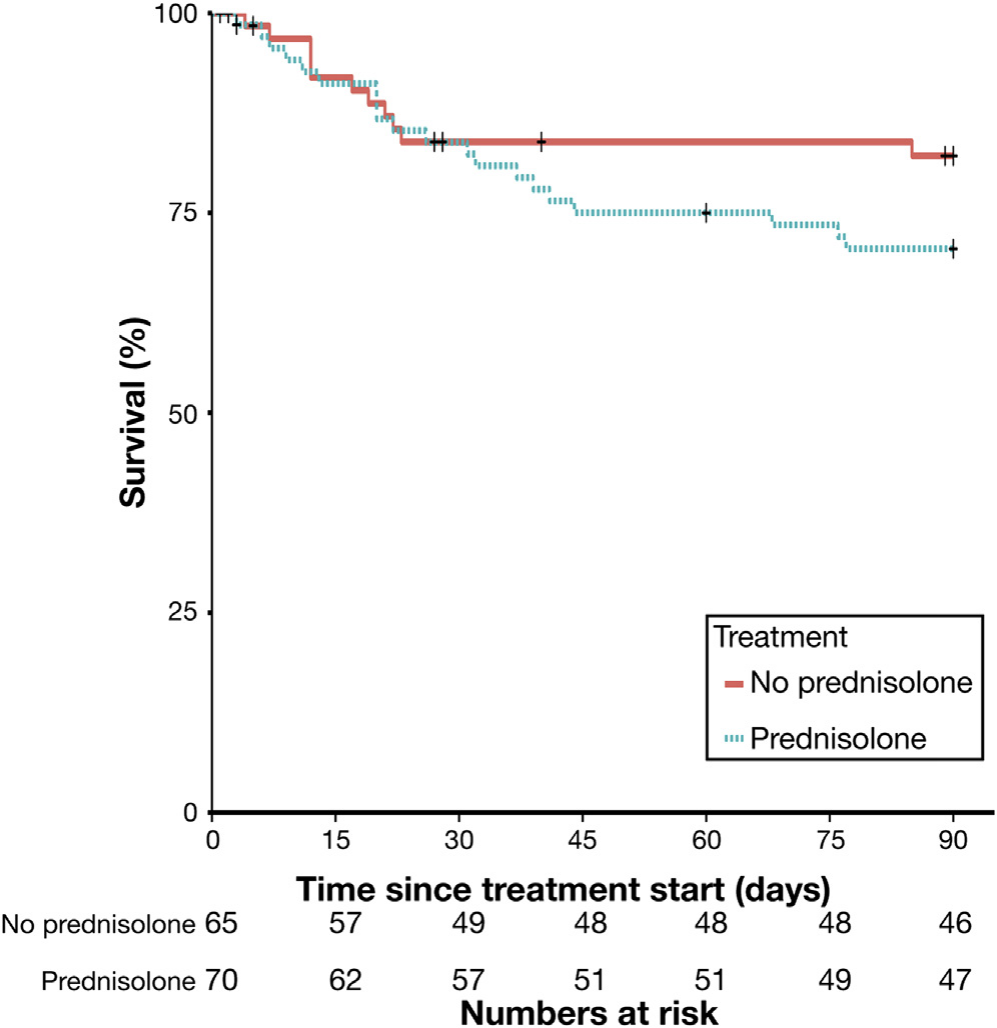
Comparison of survival curves to 90 days in patients with matched and high bDNA levels who were treated with prednisolone vs no prednisolone.

Finally, the strategy of using ^hi^bDNA to exclude use of prednisolone was modeled by matching ^hi^bDNA patients in the prednisolone-treated group with patients in the no-prednisolone group. This would estimate the likely mortality at 90 days if these patients had not received prednisolone. In patients with ^hi^bDNA, avoidance of prednisolone treatment was associated with a reduction in 90-day mortality (17% vs 29%; OR, 1.96; 95% CI, .84−4.3; *P* = .05) (Figure 3).

## Discussion

Our analysis of 1092 patients with SAH confirms that infection is highly prevalent, with 12% having infection at baseline and 23% of SAH patients developing infection on treatment. Prednisolone is associated with a significant increase in the risk of serious infections.^3^ Furthermore, these data indicate that prednisolone therapy appears to confer an excess risk of post-treatment infections, irrespective of severity. Cabre et al^14^ also described an increased rate of late infections in patients treated with prednisolone compared to those treated with enteral nutrition. This phenomenon might partly explain why early improvements in liver function attributable to prednisolone did not translate into a sustained survival benefit.

Although there was no overall association between presentation with infection and mortality, these data suggest that baseline infection might not be entirely benign. Decisions regarding continuation of antibiotic therapy are important when patients are to receive prednisolone. The current study suggests that continued antibiotic therapy in patients with baseline infection confers a survival advantage.

The impact of infection on 90-day mortality is critically modulated by prednisolone. In patients treated with prednisolone, infection exerts an independent effect on mortality by 90 days. When prednisolone is not used, the effect of infection on 90-day mortality is secondary to baseline liver impairment and early improvement in liver function. In other words, patients who are not treated with prednisolone but who have poor liver function are more likely to develop infection and die within 90 days. Further, we show that development of infection before calculation of the Lille score at day 7 is associated with classification as a Lille nonresponder; this timing raises the possibility that early infection might modulate Lille score. In patients who developed infection within the first 7 days, prednisolone dramatically increased the risk of mortality at 90 days.

Concerns about infectious complications have restricted use of prednisolone. As a result, strategies that aim to first test for benefit from prednisolone before continued use have gained support. One approach is to use the Lille model after 7 days of prednisolone therapy to determine whether corticosteroids should be continued or not. However, in a trial of patients who were Lille nonresponders after 7 days of corticosteroid therapy, there was no survival benefit associated with withdrawal of prednisolone and replacement with pentoxyfilline compared with patients who were treated for the full 28 days with prednisolone.^15^ We speculate that 7 days of prednisolone therapy may be enough to impair host immunity to allow development of serious infection, and that discontinuation of steroids after 7 days may be unable to reverse the damage.

Consequently, the ability of pretreatment bDNA levels to predict the development of infection in patients who were uninfected at the time of sampling and who subsequently receive prednisolone is of interest. This strategy differs from previous studies in which investigators sought to differentiate SAH patients with infection *at the time of presentation* from those without,^16^ and is the first attempt to evaluate bDNA in the context of corticosteroid immunosuppression.^17^ While the area under the receiver operating characteristic for bDNA to predict the subsequent development of infection was modest in the current study, bDNA was nonetheless superior to white blood cell count in this regard. Also of interest is the observation that bDNA was not predictive of infection when patients were not subsequently treated with prednisolone: only when the immune system had been modulated by prednisolone *and* when the circulating bacterial load was high was there a heightened risk of developing infection. The ability of bDNA to predict infection before alternative immunosuppressive agents are used is an enticing prospect that warrants dedicated testing.

bDNA level may also be regarded as a target for therapy before initiation of immunosuppression. Where culture results are unavailable but bDNA levels are high, a possible paradigm could be to repeat microbiological screening and treat with broad-spectrum antibiotics until bDNA has returned to normal levels. Randomly allocated empirical broad-spectrum antibiotic therapy in SAH is the subject of ongoing clinical trials.^18^, ^19^

The translocation of bacterial products from gut to portal vein in heavy alcohol drinkers has been proposed as a mechanism of hepatic injury and cause of hepatic inflammation in SAH.^20^ Indeed, >90% of SAH patients had detectable bDNA levels in the current study, which is substantially higher than rates seen in healthy controls, patients with suspected bloodstream infections, and patients with other forms of decompensated liver disease.^17^, ^21^, ^22^ The higher rate of bacteremia seen in these SAH patients might represent extensive bacterial translocation^23^ or defective leukocyte clearance,^24^, ^25^, ^26^ or both. Bacterial translocation has been implicated in the pathogenesis of SAH.^20^, ^27^ However, in the current study, while circulating bDNA predicted the development of infection, it did not correlate with markers of baseline liver function such as MELD, DF, or Glasgow Alcoholic Hepatitis Score.

In common with other studies in the field, the central limitation of this study is the lack of a gold standard to diagnose infection. In our data, only a minority of infections (40% of incident infections) yielded an organism on microbiological culture; most were diagnosed clinically. Clinical diagnosis of infection will be sensitive but may lack specificity, with physicians unable to differentiate inflammatory responses driven by underlying alcoholic hepatitis, from infection. However, in this regard, we highlight the contrasting outcomes of patients diagnosed with infection in this study in relation to the double-blind allocation of prednisolone. The association between randomly allocated prednisolone therapy and poor outcomes for this subset of patients suggests that they had a condition exacerbated by immunosuppression, which is very likely to have been infection.

No treatment was shown to reduce 90-day mortality for SAH in the STOPAH study.^3^ In the current retrospective analysis, a reduction in 90-day mortality was estimated by using pretreatment bDNA level to guide prescription of prednisolone and was of borderline statistical significance. Larger prospective randomized studies are needed to definitely report whether bDNA-guided therapy can impact on mortality, in SAH, and perhaps in other acute inflammatory conditions where immunosuppression is required.

In summary, these data show that infections are frequent in SAH, but are only independently associated with mortality when patients receive prednisolone. These infections may be predicted by measuring levels of circulating bacterial DNA, raising the possibility that such infections, and consequent mortality, could be avoided by bDNA-stratified prednisolone prescribing for SAH patients.

## Figures and Tables

**Supplementary Table 1:** Development of Infection and Death in Each of the Study Periods: at Baseline, on Treatment and Post-Treatment

Variable treatment arm	Baseline infection		Incident infection			
			On treatment		Post-treatment	
	Prednisolone (n = 547)	No prednisolone (n = 545)	Prednisolone (n = 547)	No prednisolone (n = 545)	Prednisolone (n = 463)	No prednisolone (n = 447)
Deaths	NA	NA	76 (14)	98 (18)	145 (31)	141 (32)
Patients developing an infection	64 (12)	71 (13)	127 (23)	124 (23)	56 (12)	33 (7)
Patients developing infection at >1 site	2 (0.4)	4 (0.7)	8 (2)	10 (2)	6 (1)	4 (0.9)
Patients developing >1 infection	0	0	16 (3)	12 (2)	0	0

NOTE. Values are n (*%*). Subgroups of patients developing more than 1 infection or at more than 1 site are shown.

NA, not applicable.

**Supplementary Table 2:** Full Breakdown of Infections by Site and Time of Occurrence in Relation to Presentation and Treatment

Site of infection	Baseline infection (n = 141)	Incident infection	
		On treatment (n = 301)	Post-treatment (n = 102)
Respiratory, n (*%*)	42 (33.6)	110 (36.5)	40 (39.2)
Lower respiratory tract, n	41	108	39
Upper respiratory tract, n	1	2	1
SBP and bacteremia, n (*%*)	28 (22.4)	75 (24.9)	23 (22.5)
SBP, n	16	47	13
Bacteremia, n	8	28	10
Urinary, n (*%*)	24 (19.2)	31 (10.3)	10 (9.8)
Other, n (*%*)	12 (9.6)	46 (15.3)	10 (9.8)
Biliary, n	1	0	0
Intra-abdominal, n	1	6	2
Deep tissue, n	1	4	1
Gastrointestinal, n	3	9	3
Skin and soft tissue, n	6	25	2
Orodontal, n	0	2	0
ENT, n	0	0	2
Unknown, n (*%*)	19 (15.2)	36 (12.0)	18 (17.6)
Missing data	14	3	1

ENT, ear, nose, and throat; SBP, spontaneous bacterial peritonitis.

**Supplementary Table 3:** Full Breakdown of Organisms Isolated From Patients Presenting With or Developing Infection, by Relation in Terms of Timing to the Treatment Period

Organism	Baseline	Incident infection	
		On treatment	Post-treatment
Gram-negative bacilli, n (*%*)	23 (57.5)	45 (42.9)	19 (67.9)
*Campylobacter* spp, n	0	1	
Coliforms (NOS), n	5	4	1
*Enterobacter* spp, n	0	1	
*Enterobacter cloacae*, n	1	1	
*Escherichia coli*, n	12	25	8
*Fusobacterium nucleatum*, n	0	0	1
*Klebsiella* spp, n	1	0	1
*Klebsiella pneumoniae*, n	2	7	4
*Pseudomonas* spp, n	0	2	
*Serratia marcescens*, n	0	1	1
Unknown, n	2	3	3
Gram negative coccus, n (*%*)	0	2 (1.9)	0
*Moraxella catarrhalis*, n	0	1	
Unknown, n	0	1	
Gram positive bacilli, n (*%*)	2 (5)	7 (6.7)	3 (10.7)
*Clostridium difficile*, n	2	6	3
Unknown, n	0	1	
Gram positive coccus, n (*%*)	11 (27.5)	44 (41.9)	5 (17.9)
*Enterococcus* spp, n	2	9	1
*Enterococcus faecalis*, n	1	1	
*Enterococcus faecium*, n	0	2	
*Gemella* spp, n	0	1	
*Micrococcus* spp, n	0	1	
*Staphylococcus* spp, n	2	6	1
*Staphylococcus aureus*, n	1	7	3
*Staphylococcus epidermidis*, n	0	1	
*Staphylococcus warneri*, n	0	1	
*Streptococcus* spp, n	2	9	
*Streptococcus anginosus*, n	1	0	
*Streptococcus gordonii*, n	1	0	
*Streptococcus mitis*, n	0	1	
*Streptococcus pneumoniae*, n	1	1	
*Streptococcus viridans*, n	0	1	
Unknown, n	0	3	
Mixed NOS, n (*%*)	2 (5)	2 (1.9)	0
Fungus, n (*%*)	2 (5)	4 (3.8)	1 (3.6)
*Candida* spp, n	1	0	
*Candida albicans*, n	0	4	1
Unknown, n	1	0	
Viral, n (*%*)	0	1 (1.0)	0
Norovirus, n	0	1	

NOS, not otherwise specified.

**Supplementary Table 4:** Baseline Characteristics of Bacterial DNA Cohort, by Day 7 Infection Status

Variable	No early-onset infection (n = 638)	Early-onset infection (n = 93)
Age, *y*	49 (42−57)	50 (41−58)
Sex, *male*, n (*%*)	415 (57)	54 (58)
Alcohol consumption, *U/wk*	126 (81−206)	140 (98−213)
Prednisolone, n (*%*)	326 (51)	40 (43)
Systolic blood pressure, *mm Hg*	111 (103−121)	110 (100−123)
Diastolic blood pressure, *mm Hg*	68 (60−75)	65 (60−72)
Pulse, *beats/min*	90 (80−98)	92 (80−101)
Temperature, *°C*	36.8 (36.5−37.1)	36.8 (36.5−37.0)
Haemoglobin, *g/L*	108 (95−121)	100 (90−114)
White blood cell count, *×10*^*3*^*per mm*^*3*^	8.7 (6.0−12.3)	10.3 (7.3−14.7)
Neutrophils, *×10*^*3*^*per mm*^*3*^	5.9 (4.0−9.3)	7.9 (5.4−12.0)
International normalised ratio	1.7 (1.5−2.0)	1.9 (1.6−2.2)
Albumin, *g/dL*	24 (21−29)	25 (21−29)
Bilirubin, *mg/dL*	16.5 (10.0−24.3)	17.3 (11.7−24.6)
Alanine transaminase, *IU/L*	44 (31−64)	41 (26−66)
Aspartate transaminase, *IU/L*	128 (90−176)	120 (86−155)
Sodium, *mmol/L*	134 (130−137)	133 (129−136)
Urea, *mmol/L*	3.2 (2.2−5.1)	3.7 (2.3−5.6)
Creatinine, mg/dL	0.72 (0.60−0.95)	0.74 (0.58−1.09)
Discriminant function	54 (42−71)	61 (46−81)
Model for End-Stage Liver Disease	23 (21−26)	24 (22−29)
Pretreatment antibiotics,^a^ n (*%*)	156/638 (24)	39/93 (42)
bDNA, *pg/mL*	9.2 (3.2−23.8)	12.3 (5.6−39.4)

NOTE. Data are median (interquartile range) unless other indicated.

MELD, Model for End-Stage Liver Disease.

a Pretreatment antibiotics defined as intravenous antibiotics commenced within 5 days prior to starting trial therapy.

**Supplementary Table 5:** Area Under Receiver Operating Curve Comparison Between Bacterial DNA and White Blood Cell Count

Parameter	AUROC	*P* value	95% CI
bDNA	0.704	.003	.57−.83
White blood cell count	0.577	.265	.44−.72

AUROC, area under receiver operating curve.

**Supplementary Table 6:** Effect of Smoking on Risk of Developing Incident Infection

Term	OR (95% CI)	*P* value
Smoking (never)	Reference	.14
Smoking (current)	.68 (.46−1.01)	.06
Smoking (prior)	.91 (.58−1.41)	.67
Prednisolone	1.23 (.88−1.73)	.23

## Supplementary Results

### Alcohol and Infection Risk

Drinking data was collected at the day-90 visit time point. Data regarding drinking behavior are incomplete and available for 478 patients surviving to complete visit at 90 days. For the purposes of subsequent analysis, drinking behavior is considered dichotomously as abstinent or drinking.

There is no association between prednisolone treatment and a return to drinking (*P* = .95) in the cohort of patients with available data. No association is revealed when analysis is restricted to those completing treatment (survival to day 28) or surviving a minimum of 90 days after the start of treatment.

No association is revealed between the reported maximum level of alcohol consumption at baseline and the development of a serious infection (SAE) (*P* = .89), on treatment (*P* = .11), or post-treatment (*P* = .20) infection. There was a trend toward significance for an increased risk of post-treatment infection associated with a return to drinking (OR, 1.68; 95% CI, 0.97−2.91; *P* = .07). This result should, however, be interpreted with caution. It is highly probable that patients who had returned to drinking and developed an infection were more likely to attend follow-up than those who had returned to drinking but experienced no infection. Follow-up visits may, for example, have been completed opportunistically when patients who had returned to drinking had attended hospital for assessment and treatment of their infective complication. Consequently, the sample of patients who returned to drinking might well be biased in favor of those who had developed an infection.

### Smoking and Infection Risk

Smoking data were available on 986 subjects. A minority of patients reported never smoking (n = 306 [31%]), while the remainder were split between active (n = 447 [41%]) and former smokers (n = 233 [21%]).

No statistically significant effect of smoking status (never, current, former) was found on the occurrence of chest infections, including when adjusted for prednisolone treatment (Supplementary Table 6).

### Bacterial DNA Analyses

Whole blood samples were available for bDNA analysis in 68% (740 of 1092) of patients included in the clinical data analysis. There was insufficient sample available from the remaining patients (352 of 1092 [32%]) who participated in the STOPAH study. In an additional 9 patients, the discrepancy between PCR replicates was >1 copy cycle and the result deemed invalid. A bDNA result was therefore not available in 361 of 1092 patients (33%). Incident infection in patients for whom bDNA was measured was higher than in patients for whom bDNA was not measured (228 of 731 [31%] vs 81 of 362 [22%]), although the mortality rate within the population in whom bDNA was measured was similar to the mortality rate for those in whom bDNA was not measured (29% [106 of 361]) vs 25% [180 of 731]).
